# Prevalence of needlestick injury among healthcare workers in Ethiopia: a systematic review and meta-analysis

**DOI:** 10.1186/s12199-019-0807-7

**Published:** 2019-08-14

**Authors:** Teshiwal Deress Yazie, Kasaw Adane Chufa, Mekonnen Girma Tebeje

**Affiliations:** Unit of Quality Assurance and Laboratory Management, School of Biomedical and Laboratory Sciences, College of Medicine and Health Sciences, University of Gondar, P.O. Box 196, Gondar, Ethiopia

**Keywords:** Needlestick injury, Percutaneous exposure, Occupational exposure, Healthcare worker, Ethiopia

## Abstract

**Background:**

Health facilities can provide diagnostic, curative, and prognostic services for the community. While providing services, healthcare workers can be exposed to needlestick injuries that can transmit pathogenic organisms through body fluids.

**Objective:**

The aim was to establish the pooled prevalence of needlestick injuries among healthcare workers in Ethiopia.

**Methods:**

This systematic review and meta-analysis was conducted according to PRISMA guidelines. Articles were searched from Google Scholar, PubMed, Science Direct, and Scopus databases using a combination of keywords and Boolean functions. All the searched articles were imported into the EndNote X9 software, and then, duplicate data files were removed. Article screening and data extraction were done independently by two authors. Data manipulation and analyses were done using STATA version 15.1 software.

**Results:**

The analysis of 23 full-text articles showed that the prevalence of the 12-month and lifetime needlestick injuries among the primary studies ranged from 13.2 to 55.1% and 18.6 to 63.6%, respectively. The pooled prevalence of needlestick injuries among the Ethiopian healthcare workers was 28.8% (95% CI 23.0–34.5) and 43.6% (95% CI 35.3–52.0) for the 12 months and lifetime, respectively.

**Conclusions:**

The pooled prevalence of needlestick injuries among Ethiopian healthcare workers was high. Therefore, efforts should be implemented to reduce the occurrence of injuries. Adequate protective equipment and safety-engineered devices should be supplied for the healthcare workers. It could be more effective to reduce the factors contributing to increased exposures through the allocation of adequate numbers of the healthcare workforce and implementing in-service training.

## Background

Healthcare facilities (HCFs) can provide diagnostic, preventive, curative, and prognostic services for the community. However, while they are providing services, healthcare workers (HCWs) are exposed to blood and body fluids through occupational sharps, splashes, and needlestick injuries [1, 2]. Particularly, there is a potential exposure among doctors, nurses, laboratory professionals, and biomedical waste management staff to blood-borne pathogens worldwide [3–5]. Needlestick injuries (NSIs) are the most common workplace-related health hazards responsible for the transmission of blood-borne pathogens [6, 7] among the HCWs where safety measures have not already been established [2]. Needles caused accidental penetration of the skin [2, 8–11]. Injuries mostly happen during needle recapping, operative procedures, blood sample collection, intravenous line administration, and poor waste disposal practices [12]. Following NSIs, more than 20 blood-borne pathogens can be transmitted through body fluids [11, 13]. However, the most common diseases that can be potentially transmitted through body fluids are HIV, HBV, and HCV [11].

Though currently the exact incidence of NSIs is believed to be underreported [14], the World Health Organization (WHO) reported as 3 million HCWs were exposed to blood-borne viruses each year globally. From this, 2 million, 900,000, and 300,000 were contributed to HBV, HCV, and HIV, respectively, and the majority (90%) happened in the developing countries [15, 16]. The high incidence of NSIs associated with blood-borne infections among developing countries is mainly attributed due to the high disease prevalence and lack of proper personal protective devices [17, 18]. The risk of acquiring HBV, HCV, and HIV infections from the sharp exposure when the source patient is positive can range from 2 to 40%, 3 to 10%, and 0.2 to 0.5%, respectively [19, 20]. In addition, HBV can survive up to a week under optimal conditions and has been detected from the discarded needles [21]. The morbidity and mortality associated with occupational hazards are impacting the health and productivity of the health workers [22] through high cost, health consequences, emotional distress, and missing working days [23, 24]. Currently, there is no review conducted with respect to the estimation of NSI prevalence in Ethiopia. Therefore, the aim of this systematic review and meta-analysis was to estimate the pooled prevalence of NSIs among the healthcare workers in Ethiopia.

## Methods

### Setting

Ethiopia is a highly populated country in the Horn of Africa. Though, currently, the exact number of the population is unknown, during 2012, it was predicted to be 84,320,987 [25]. Due to rapid population growth, the number of health facilities is increasing [26, 27]. Currently, the healthcare management is grouped into primary, secondary, and tertiary levels. During 2011, there were a total of 22,792 health facilities in the country. From this, hospitals, health centers, health posts, and private clinics accounted for 125, 2999, 15,668, and 4000, respectively [28]. The health posts and health centers provided basic health services to the community, and an estimated 3000–5000 and 40000 population, respectively, is allocated for them. Similarly, primary hospitals serve about 60000–10000 population. General and specialized hospitals cover a wide catchment area, and they provide specialized and referral services for about 1–5 million population [29]. Currently, with the rapid increment of HCFs, the ratio of the healthcare worker task force to the health facilities is becoming quite inadequate [30].

### Article searching strategy

Literature search, selection, data extraction, and reporting of the results were conducted according to the Preferred Reporting Items for Systematic Reviews and Meta-Analyses (PRISMA) guidelines [31]. Online electronic databases including Google Scholar, PubMed, Science Direct, and Scopus were searched using a combination of keywords and Boolean functions:

1. (needle injur* OR needlestick injur* OR percutaneous injur* OR occupation* exposure OR accident* exposure OR Body fluid* exposure OR accidental occupational exposure OR Occupational hazard*)
2. (health care worker* OR health worker* OR health staff OR medical personnel OR health personnel)
3. Ethiopia

1 AND 2 AND 3

### Eligibility criteria

Articles were included in the study only if they reported the 12-month, lifetime, or both prevalence of NSIs. Primary full-text articles published in English from the Ethiopian settings were the inclusion criteria thereby excluding letters to editors, short communications, and review articles. In addition, the aggregate report of needlestick and sharps injuries were excluded from the study.

### Study selection and data extraction

All the searched articles were imported into the EndNote version X9 software, and then, duplicate files were removed. Two investigators (TD and MG) independently screened articles by their title, abstract, and full-text to identify potentially eligible studies according to the predetermined inclusion criteria, and then, the screened articles were compiled together from the two reviewers. The data extraction form was prepared in Microsoft Excel Spreadsheet. Data were extracted from the full-text articles by two reviewers (TD and KA) independently. The data extraction form includes the name of the first author, year of publication, setting (region of the country), study group, sample size, number of needlestick injuries, 12 months prevalence, and lifetime prevalence. Any discrepancy between the two data extractors was resolved by discussion.

### Statistical analysis

The extracted data were categorized into 12 months and lifetime needlestick injury and entered into the STATA version 15.1 separately. The prevalence estimates were conducted using the metaprop program. Proportions of exposure (p) and the corresponding standard errors (se) were calculated using p = r/n and se = √p(1 − p)/n, respectively. However, to normalize the distribution, study level estimates were logit transformed using logitp = ln[p/(1-p)], and the corresponding standard error (se) of logit event estimates se = √1/r + 1/(n-r) was calculated. In situations with high across study heterogeneity, the use of random effects models is recommended [32]. The DerSimonian and Laird method is the most common method for using a random effects model for the meta-analysis [33]. The presence of heterogeneity among the studies was checked using the *I*^2^ test statistics. The *I*^2^ statistics estimates the presence of observed difference between studies due to heterogeneity, and it can range from 0 to 100%. A value of 0% indicates the absence of heterogeneity whereas 100% indicates the presence of significant heterogeneity. The 25%, 50%, and 75% values represent low, medium, and high heterogeneity between studies, respectively [34]. In addition, a *p* value of less than 0.05 is used to declare heterogeneity [35]. In this meta-analysis, in both 12 months and lifetime prevalence estimates of NSIs, the *I*^2^ values were found to be high (> 75%). Since this value is a definite indicator of significant heterogeneity, the analysis was conducted using a random effects model with 95% CI as opposed to the fixed effects model to adjust the observed variability among the studies. Moreover, the sources of heterogeneity were assessed through subgroup analysis, sensitivity analysis, and meta-regression. Finally, small study effects and publication bias were analyzed through visual inspection of the funnel plots and objectively using Egger’s test. All the data manipulations and analysis were performed using the STATA version 15.1 software.

### Quality assessment

The quality of the included studies was assessed using the Joanna Briggs Institute (JBI) quality assessment tool for the prevalence studies [36]. The evaluation criteria included nine parameters: (1) appropriate sampling frame, (2) proper sampling technique, (3) adequate sample size, (4) study subject and setting description, (5) sufficient data analysis, (6) use of valid methods for the identified conditions, (7) valid measurement for all participants, (8) using appropriate statistical analysis, and (9) adequate response rate. Two reviewers (TD and MG) assessed the quality of included studies. Finally, studies were categorized into high risk of bias and low risk of bias using 50% as a cutoff value. Articles with a score of ≥ 50% were considered as a low risk of bias.

## Results

**Fig. 1:**
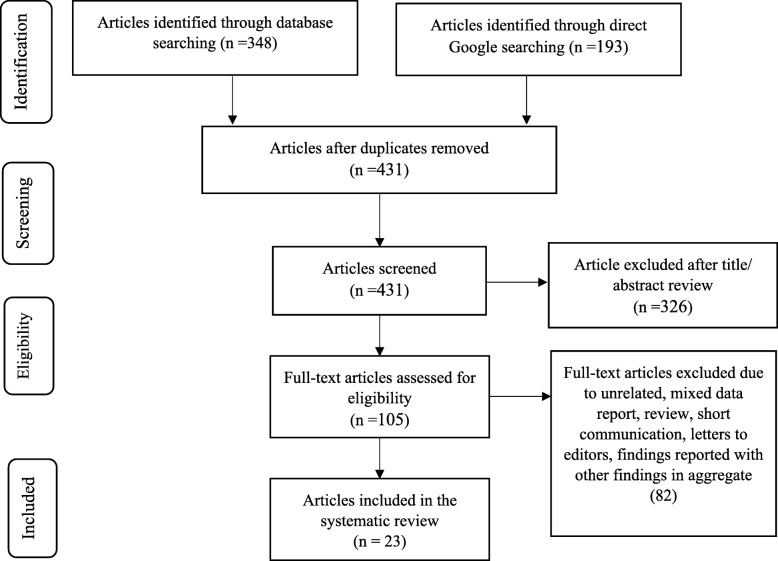
The PRISMA flow diagram showing the study selection process

All the relevant studies published from the Ethiopian settings were searched without any time restriction and manipulated according to the PRISMA guidelines [31]. A total of 348 and 193 articles were retrieved from the database and manual searching, respectively. From this, 110 articles were excluded due to duplication. The remaining 431 articles were evaluated, and 326 data files were excluded based on their title and abstract. Further, 105 full-text articles were screened and 82 were excluded due to being review articles, studies conducted on students, short communications, letters to the editors, and aggregate report of sharp and NSI data. Finally, 23 articles were included in this study (Fig. 1).

### Characteristics of included studies

A total of 23 articles [37–59] were included in this systematic review and meta-analysis, with an overall sample size of 7468 healthcare workers. All the included studies were cross-sectional studies. The earliest study was conducted during 2009 [55], and the latest two articles were published in 2019 [58, 59]. Overall information regarding the prevalence of NSIs was obtained from five regions and two self-administrative cities including Amhara [41, 44, 47, 49, 51, 53, 57–59]; Oromia [42, 48, 56]; Southern Nations, Nationalities, and People (SNNP) [37, 46, 50]; Somali [38]; and Harari [43]; two studies were conducted on both Somali and Dre Dawa (SAC) [52, 55], and four articles were obtained from two self-administrative cities Addis Ababa and Dire Dawa [39, 40, 45, 54]. The sample size across the studies was ranged from 162 [37] to 760 [46]. Among the studies, ten articles exclusively reported the 12-month NSI prevalence [38, 41, 42, 44, 50–53, 55, 56]. Similarly, seven studies exclusively reported the lifetime prevalence of the NSI [37, 39, 40, 47, 54, 57, 58]. The remaining five articles have reported both the 12-month and lifetime needlestick injury prevalence [43, 45, 46, 48, 49, 59]. The quality of each of the included studies was evaluated using a nine-item risk of bias assessment tool [36]. All studies confirmed a low risk of bias (Table 1).

**Table 1:** Characteristics of the included studies in the meta-analysis of the prevalence of needlestick injury among healthcare workers in Ethiopia, 2019

First author (reference)	Year	Region	Study group	Article quality	Sampling technique	Facility type	Sample size (*n*)	Participants with NSI	
								12 months	Lifetime
Beyene [37]	2014	SNNP	HCP	Low risk	Nonprobability	HS and HC	162		58
Mideksa [38]	2014	Somali	HCP	Low risk	Probability	HS and HC	316	95	
Desalegn [39]	2015	SAC	HCP	Low risk	Nonprobability	HS	254		155
Elfu [40]	2013	SAC	HCP	Low risk	Probability	HS	645		277
Kebede [41]	2018	Amara	HCP	Low risk	Probability	HS	258	89	
Bidira [42]	2014	Oromia	HCP	Low risk	Unknown	HS	211	83	
Reda [43]	2010	Harari	HCP	Low risk	Probability	HS and HC	475	83	145
Kebede [44]	2012	Amara	HCP*	Low risk	Probability	HS and HC	344	106	
Mekonnen [45]	2018	SAC	HCP	Low risk	Probability	HS and HC	305	81	164
Tadesse [46]	2016	SNNP	HCP	Low risk	Probability	HS and HC	760	419	483
Teju [47]	2015	Amara	HCP	Low risk	Probability	HC	194		83
Bekele [48]	2015	Oromia	HCP*	Low risk	Probability	HS	362	69	134
Azage [49]	2014	Amara	HCP	Low risk	Probability	All types	209	61	104
Kaweti [50]	2016	SNNP	HCP*	Low risk	Unknown	HS	496	132	
Dilie [51]	2017	Amara	HCP	Low risk	Probability	HS and HC	213	28	
Alemayehu [52]	2016	Harari^#^	*	Low risk	Probability	HS and HC	253	69	
Aynalem [53]	2014	Amara	HCP	Low risk	Nonprobability	HS and HC	234	74	
Taddesse [54]	2016	SAC	HCP*	Low risk	Nonprobability	HS	313		98
Reda [55]	2009	Harari^#^	HCP	Low risk	Probability	HS and HC	330	96	
Girma [56]	2015	Oromia	HCP*	Low risk	Probability	HS	232	71	
Yizengaw [57]	2018	Amara	HCP*	Low risk	Probability	HS	388		72
Adane [58]	2019	Amara	HCP	Low risk	Probability	HS	332		194
Yasin [59]	2019	Amara	HCP	Low risk	Probability	HS	282	58	119

*SAC* self-administrative cities, *HCP* healthcare professional, *Harari*^*#*^ Harari and Dire Dawa, HCP* healthcare professional and cleaner, *HS* hospital, *HC* health center, *SNNP* Southern Nations, Nationalities, and People

### Prevalence of needlestick injury

**Fig. 2:**
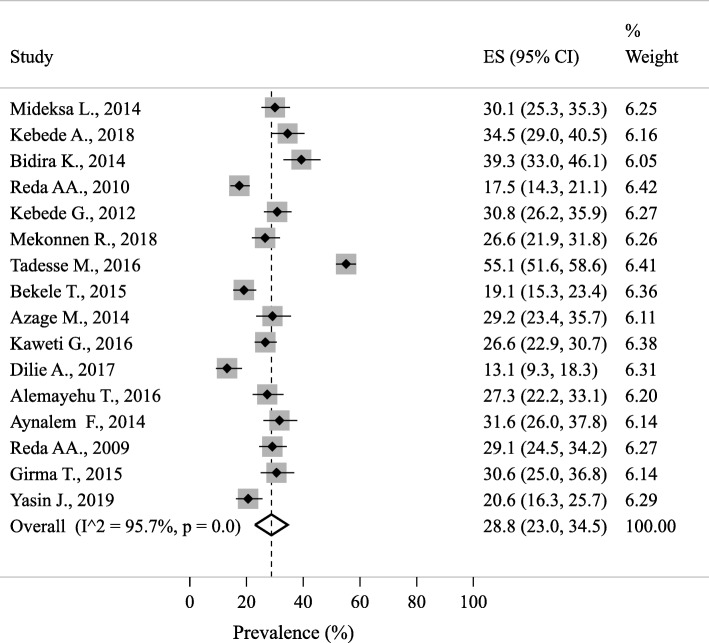
The 12-month prevalence of needlestick injuries among the Ethiopian healthcare works

The prevalence of 12 months NSI among the Ethiopian HCWs was ranged from 13.2% in Amhara region [51] to 55.1% in the SNNP region [46]. The 12-month pooled prevalence of NSIs using the random effects model was 29.3% (95% CI 23.3–35.4; Fig. 2).

**Fig. 3:**
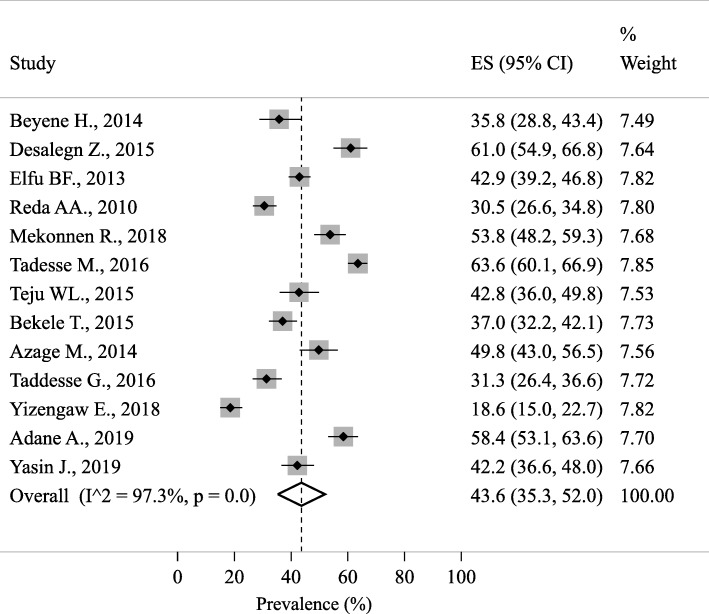
The lifetime pooled prevalence of needlestick injuries among the Ethiopian healthcare workers

The lifetime NSI prevalence was ranged from 18.6% in Amhara region [57] to 63.6% in the SNNP region [46]. The lifetime pooled prevalence of NSI among the Ethiopian HCWs was 43.6% (95% CI 35.3, 52.0; Fig. 3).

### Investigation of heterogeneity

Heterogeneity in meta-analysis is inevitable due to differences in study quality, methodology, sample size, and sampling technique among the studies. The included studies in this meta-analysis exhibited high level of heterogeneity (*I*^2^ = 95.7%, *p* < 0.001, and *I*^2^ = 97.3%, *p* < 0.01) for the 12-month and lifetime NSI prevalence estimates, respectively. So, the random effects model was used to adjust the observed variability. To identify the possible source of heterogeneity, subgroup analyses were carried out based on the year of publication, sampling technique, setting, facility type, and study groups. However, the level of heterogeneity remained high after subgroup analysis (Table 2). In addition, a sensitivity test was done to identify the influence of each study and the result indicated no influence on the pooled estimate while removing one study at a time from the analysis.

**Table 2:** Subgroup analysis of the prevalence of 12 months and lifetime needlestick injuries among the Ethiopian healthcare workers, 2019

Prevalence type	Variable category	Prevalence (%)	95% CI	*p* value	*I* ^2^
A 12-month prevalence	Healthcare facility				
	Hospital	28.3	23.05, 33.5	0.001	86.2
	Hospital and HC	29.03	19.7, 38.4	0.001	97.4
	Sampling technique				
	Probability	29.06	22.3, 35.8	0.001	96
	Non-probability	22.1	19.1, 25.1		
	Study group				
	HCPs	31.02	21.6, 40.4	0.001	97.0
	HCPs and cleaners	25.54	20.3, 30.7	0.001	87.0
	Year of publication				
	2009–2014	29.42	23.8, 35.0	0.001	88.14
	2015–2019	29.12	18.8, 39.4	0.001	97.52
	Geographical location				
	Amhara region	26.51	19.6, 33.4	0.001	90.35
	Oromia region	29.45	17.4, 41.5		
	Others	30.34	19.8, 40.8	0.001	97.6
Lifetime prevalence	Healthcare facility				
	Hospital	41.6	30.4, 52.7	0.001	97.3
	Hospital and HC	46.0	28.5, 63.5	0.001	98.1
	Sampling technique				
	Probability	45.4	34.9, 55.9	0.001	97.7
	Non-probability	39.6	25.9, 53.4	0.001	96.0
	Study group				
	HCPs	48.1	40.4, 55.9	0.01	95.6
	HCPs and cleaners	28.9	17.4, 40.3		
	Year of publication				
	2009–2014	39.6	31.4, 47.9	0.001	90.2
	2015–2019	45.4	32.8, 57.0	0.001	98.0
	Geographical location				
	Amhara region	42.3	26.2, 58.3	0.001	97.6
	Oromia region	43.0	32.1, 53.9	0.001	94.9
	Others	46.0	28.5, 63.5	0.001	98.1

Others include Harari, SNNP, Somali, and Dire Dawa. *HC* health center

In addition, we tried to investigate the possible sources of heterogeneity through meta-regression using sample size and year of publication as covariates. Meta-regression is a preferable method of investigating heterogeneity than subgroup analysis and has the advantage of running multiple covariates simultaneously [60]. The result of the meta-regression analysis indicated that the variables were not significantly associated with the presence of heterogeneity for both 12 months and lifetime prevalence estimates (Table 3). Further, a sensitivity analysis was conducted; however, in both cases, there was no single study influence on the pooled prevalence estimates of NSIs.

**Table 3:** A meta-regression analysis of factors for heterogeneity of the prevalence of needlestick injury among the healthcare workers in Ethiopia, 2019

Prevalence estimate	Heterogeneity source	Coefficients	Std. error	*p*-value
12 months	Publication year	0.0066458	0.0442915	0.883
	Sample size	0.0013003	0.0008385	0.145
Lifetime	Publication year	0.0471669	0.069964	0.515
	Sample size	0.0005878	0.0010243	0.579

### Publication bias

**Fig. 4:**
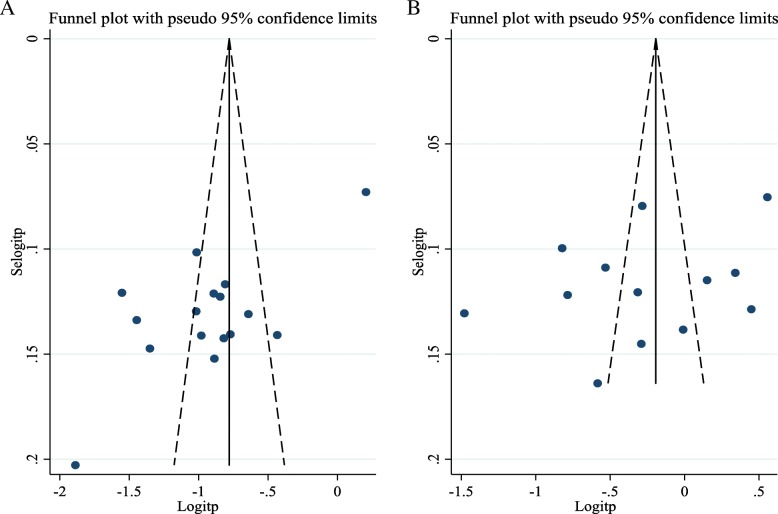
Funnel plots of the 12-month (**a**) and lifetime prevalence estimates (**b**) of the needlestick injury among the Ethiopian healthcare workers

The presence of publication bias was evaluated using funnel plots (Fig. 4) and Egger’s test. Each point in funnel plots represents a separate study, and asymmetrical distribution is evidence of publication bias [61]. First, studies’ effect sizes were plotted against their standard errors and the visual evaluation of the funnel plots indicated that there were publication biases for the 12-month prevalence estimate as the graph appears asymmetrical. The lifetime prevalence estimate was visually symmetrical. The subjective evidence of the publication bias was confirmed using Egger’s weighted regression statistics. According to the symmetry assumption, there was publication bias in the 12-month prevalence (*p =* 0.001), whereas the *p* value (0.222) was high for the lifetime prevalence estimate which declares the absence of heterogeneity among the included studies.

## Discussion

Workplace health and safety is vital in every organization, particularly in healthcare settings. However, currently, HAIs and the emergence of drug-resistant organisms are increasingly challenging. Healthcare workers in developing countries are frequently exposed to work-related injuries and become at risk of infection. Needlestick injury is one of the ways that can expose HCWs to infectious agents.

The prevalence of NSIs differs from country to county even it can vary within a country. In Ethiopia, the 12-month NSI prevalence among the primary studies ranged from 13.1 [51] to 55.1% [46]. Similarly, the lifetime prevalence ranged between 18.6 [57] and 63.6% [46]. The lifetime prevalence range was slightly better than the finding from Pakistan (30 to 73%) [62]. Also, a systematic review from Iran has estimated the NSI prevalence to be between 10 and 84.3% [63]. This variation could be due to differences in awareness, training opportunity, degree of exposure to needles, availability, and utilization of protective devices recall bias and slight methodological differences among studies. The prevalence can vary from facility to facility depending on standards, workload overload, overcrowding, type of profession and level of skills, and accessibility and use of resources. Though the lifetime prevalence may not provide a reliable prevalence estimate due to recall bias, we tried to compare the result with other studies elsewhere.

In this study, the lifetime NSI pooled prevalence (43.6%) was comparable with studies found from India (40% and 45%) [64, 65], Iran (42.5%) [66], Nigeria (46.0%) [67], Saudi Arabia (46%) [68], and Pakistan (45%) [69]. However, very high prevalence estimates were found from Pakistan (77%) [70], Iran (76%) [71], and India (68.3%) [72]. The high prevalence of the 12-month NSI from the mentioned countries could be due to the lack of training on occupational health and infection prevention or it might be due to the lack of adequate and/or proper personal protective device. Regarding the 12 months of pooled prevalence, the result in the current study (28.8%) was higher than the finding from Nigeria (9.8%) [67]. Slightly comparable results were found from Germany (31.4%) [73] and India (34% and 35.3%) [65, 74]. However, high prevalence estimate was found from India (37.5%) [72] and Iran (54%) [71]. In most cases, the result was better than the prevalence estimates from other countries. There could be a number of factors that can determine needlestick injury prevalence among countries including training, accessibility and use of proper protective devices, workload overload, working hours, recall bias, consciousness of the HCWs, and infection prevention and control strategy difference which could be the possible reasons for the variability between the pooled prevalence in the current study and the prevalence estimate from elsewhere.

With respect to subgroup analysis, the pooled prevalence for both 12 months and lifetime estimates was decreased among studies conducted using none probability sampling techniques. Similarly, in both cases, the least prevalence was obtained from the Amhara region. This difference could be due to the lack of equal resource and/or training distribution among regions or work overload difference among the HCWs. Disappointingly for both cases, a high prevalence of NSIs was obtained among HCWs alone than studies conducted on both HCWs and cleaners. Although it is difficult to provide an empirical explanation for this unexpectable finding, one can ask if overqualification leads to ignorance for safety practices. On the other hand, the heterogeneity level was not significantly decreased among different subgroups. For this reason, the possible source of variabilities could be other sources.

The current study incurred a number of limitations that are worth considering. The included studies may not allow causal relationships to be established between the outcome and predictor variables. In addition, because the primary studies were conducted based on self-reported data, they might be prone to recall bias, and as a result, the findings from the studies could likely be underreported. Further, more than one third of the studies were obtained from one region (Amhara); however, there was no study obtained from the Benshangul Gumuz, Afar, and Tigray regions. This could probably affect the generalizability of the findings at a national level. Nevertheless, the findings can provide some kind of information on the occupational exposure of HCWs to NSI in Ethiopia. It will be also important for the design and development of the appropriate strategies and interventions to reduce the high pooled prevalence of NSI in Ethiopia.

## Conclusions

The result of this study revealed that the pooled prevalence of NSI among Ethiopian HCWs was high. The inadequate allocations of HCWs among the health facilities might result in a high patient-to-staff ratio that leads to HCWs to work more hours than established standards and become more susceptible to injury. Therefore, the current study indicates the need to establish the safety and well-being of HCWs. The incidence of NSIs could be prevented by using protective equipment and safety-engineered devices. However, it could be more effective by reducing the factors that can contribute to the increased exposure of the HCWs through the allocation of adequate number of HCWs and implementing in-service training to promote standard precautions for preventing the transmission of blood-borne infections.

## Data Availability

All the data generated or analyzed during this study are included in this published article.

## Abbreviations

CI: Confidence interval
DF: Degree of freedom
HAI: Hospital-acquired infection
HC: Health center
HCFs: Healthcare facilities
HCWs: Healthcare workers
HS: Hospital
NSI: Needlestick injury
SAC: Self-administrative city
SNNP: Southern nations, Nationalities, and People
WHO: World Health Organization
